# The Impact of an Electronic Health Record Intervention on Spirometry Completion in Patients with Chronic Obstructive Pulmonary Disease

**DOI:** 10.1080/15412555.2022.2049736

**Published:** 2022

**Authors:** Christine Wang, Jonathan Siff, Peter J. Greco, Edward Warren, J. Daryl Thornton, Yasir Tarabichi

**Affiliations:** a School of Medicine, Case Western Reserve University, Cleveland, OH, USA; b Center for Clinical Informatics Research and Education, The MetroHealth System, Cleveland, OH, USA; c The Population Health Research Institute, The MetroHealth System, Cleveland, OH, USA; d Division of Pulmonary, Critical Care, and Sleep Medicine, The MetroHealth System, Cleveland, OH, USA; e Center for Reducing Health Disparities, The MetroHealth Campus of Case Western Reserve University, Cleveland, OH, USA

**Keywords:** Chronic obstructive pulmonary disease, electronic health record, informatics, pulmonary function testing, spirometry

## Abstract

Spirometry is necessary to diagnose chronic obstructive pulmonary disease (COPD), yet a large proportion of patients are diagnosed and treated without having received testing. This study explored whether the effects of interventions using the electronic health record (EHR) to target patients diagnosed with COPD without confirmatory spirometry impacted the incidence rates of spirometry referrals and completions. This retrospective before and after study assessed the impact of provider-facing clinical decision support that identified patients who had a diagnosis of COPD but had not received spirometry. Spirometry referrals, completions, and results were ascertained 1.5 years prior to and 1.5 years after the interventions were initiated. Inhaler prescriptions by class were also tallied. There were 10,949 unique patients with a diagnosis of COPD who were eligible for inclusion. 4,895 patients (44.7%) were excluded because they had completed spirometry prior to the cohort start dates. The pre-intervention cohort consisted of 2,622 patients, while the post-intervention cohort had 3,392. Spirometry referral rates pre-intervention were 20.2% compared to 31.6% post-intervention (*p* < 0.001). Spirometry completion rates rose from 13.2% pre-intervention to 19.3% afterwards (*p* < 0.001). 61.7% (585 of 948) had no evidence of airflow obstruction. After excluding patients with a diagnosis of asthma, 25.8% (126 of 488) patients who had no evidence of airflow obstruction had prescriptions for long-acting bronchodilators or inhaled steroids. A concerted EHR intervention modestly increased spirometry referral and completion rates in patients with a diagnosis of COPD without prior spirometry and decreased misclassification of disease.

## Introduction

Chronic obstructive pulmonary disease (COPD) is a leading cause of morbidity and mortality worldwide [1]. The Global Initiative for Chronic Obstructive Lung Disease (GOLD) requires spirometry for the diagnosis and management of COPD [2]. Despite this guidance, clinicians continue to diagnose COPD without obtaining spirometry. In the US, only approximately one third of patients with a new COPD diagnosis undergo spirometry [3, 4]. Without spirometry, patients risk being misdiagnosed or overdiagnosed [5–14]. In data from the multinational Burden of Obstructive Lung Disease (BOLD) study, a majority of patients (61.9%) with a physician-reported diagnosis of COPD had unobstructed postbronchodilator spirometry [15].

Overdiagnosis of COPD is not without adverse effects. Even in patients with COPD, overtreatment with inhaled anticholinergics could portend an increased risk of myocardial infarction and cardiovascular death, and overprescribing inhaled corticosteroids has been associated with an increased risk of pneumonia and osteoporosis [16–19]. In the absence of evidence-based justifications for therapy, overtreatment with long-acting inhalers can lead to unjustifiable medication side effects and significantly increases healthcare costs [16, 20].

While numerous studies have highlighted the prevalence of diagnoses of COPD without spirometric confirmation, few have demonstrated successful systematic and low-maintenance approaches to mitigate this [6, 21]. Many electronic health record (EHR) platforms have clinical decision support systems or reminder tools that support routine patient care and preventative health initiatives. These tools could conceptually be used to identify and address patients who are at risk for COPD overdiagnosis, but their efficacy in this context has not been studied. In March of 2017, we implemented a multi-pronged EHR-based intervention to address potential COPD overdiagnosis in our healthcare system. Through this study, we sought to determine the resultant impact of provider and patient directed EHR interventions on spirometry referrals and completions in our patient population.

## Methods

### Study design and setting

This retrospective before and after study was conducted at an urban, academic safety-net healthcare system that has over 18 ambulatory centers across Cuyahoga County in the state of Ohio with over 1.3 million outpatient visits per year.

### Participants

We included patients who had at least 1 ambulatory primary care encounter in the preceding 2 years and either a problem list diagnosis or an encounter-based diagnosis of COPD within the last year. COPD was defined using International Disease Classification (ICD) 10 codes of J41–J44 [22]. Patients who met inclusion criteria during an ambulatory primary care visit in the departments of Internal Medicine, Family Medicine, Medicine-Pediatrics, or Geriatrics between March 23, 2015 and September 23, 2016 were assigned to the pre-intervention group and patients meeting inclusion criteria during a visit between March 24, 2017 and September 24, 2018 were assigned to the post-intervention group. The date of the outpatient primary care visit during which patients met criteria for inclusion was regarded as the index encounter (time zero). Patients with any spirometry completed (within the institution or as ascertained from claims or health-information exchange data) prior to the start date of their respective cohort were excluded.

### Interventions

Three EHR-based interventions were implemented in March of 2017 to address the issue of COPD misdiagnosis. First, a health maintenance reminder was created to prompt primary care providers to consider spirometry testing for patients assigned a diagnosis of COPD but who had not previously had spirometry testing. This was embedded into an existing reminder list that is routinely accessed by providers and patients (*via* the patient portal) which also included timed and potentially recurring vaccination and cancer screening reminders. Second, the existing spirometry referral order was revised to make it easier for any provider to electronically and directly schedule spirometry without necessitating a phone call. Third, patients without prior spirometry testing received messages directly through the patient portal which provided them the ability to directly self-schedule spirometry.

### Data collection

All data was extracted from the Epic EHR (Epic Corporation, Verona, WI). Demographic data included age, sex, weight, race, and ethnicity. Comorbid conditions were determined using appropriate ICD 10 codes assigned up to 10 years prior to the cohort start date (ICD 10 J45 for asthma, J30 – J32 for sinus disease and I20–I28, I30–I39, I40– I52 for cardiovascular disease). Pulmonary function testing was ascertained using Current Procedural Terminology (CPT) codes assigned at our institution or identified *via* claims data or health information exchange. A referral to and completion of pulmonary function testing was considered only if it occurred within 6 months following a primary care visit. Airway obstruction was classified as a forced expiratory volume in one second (FEV1) to forced vital capacity (FVC) ratio (FEV1/FVC) less than the lower limit of normal (the 5^th^ percentile) following the administration of bronchodilators [23]. When post-bronchodilator testing was not completed, pre-bronchodilator values were used. Prescriptions for long-acting inhaled bronchodilators, including long acting muscarinic antagonists, long acting beta agonists, or inhaled corticosteroids, were also obtained from the EHR. Prescriptions were considered active if they were ordered within 6 months of a patient’s index visit.

### Outcomes

The primary outcomes were the rate of spirometry referrals and completions in each cohort within a six-month period from the index encounter. Secondary outcomes included the rates of obstruction in patients who completed testing.

### Analyses

Proportions were compared using the chi-squared test. A multiple variable logistic regression model was constructed from both cohorts to determine the odds for non-obstructed spirometry testing results after adjustment for patient age, sex, weight, race, ethnicity, smoking history and comorbid conditions. We also conducted a post-hoc interrupted time series analysis to determine if detected changes in rates of referral pre- and post-intervention were related to secular trends. Data analysis was accomplished using R version 3.5.1 [24]. The study was approved by the Human Subjects Division of The MetroHealth System (IRB19–00261).

## Results

**Figure 1.**
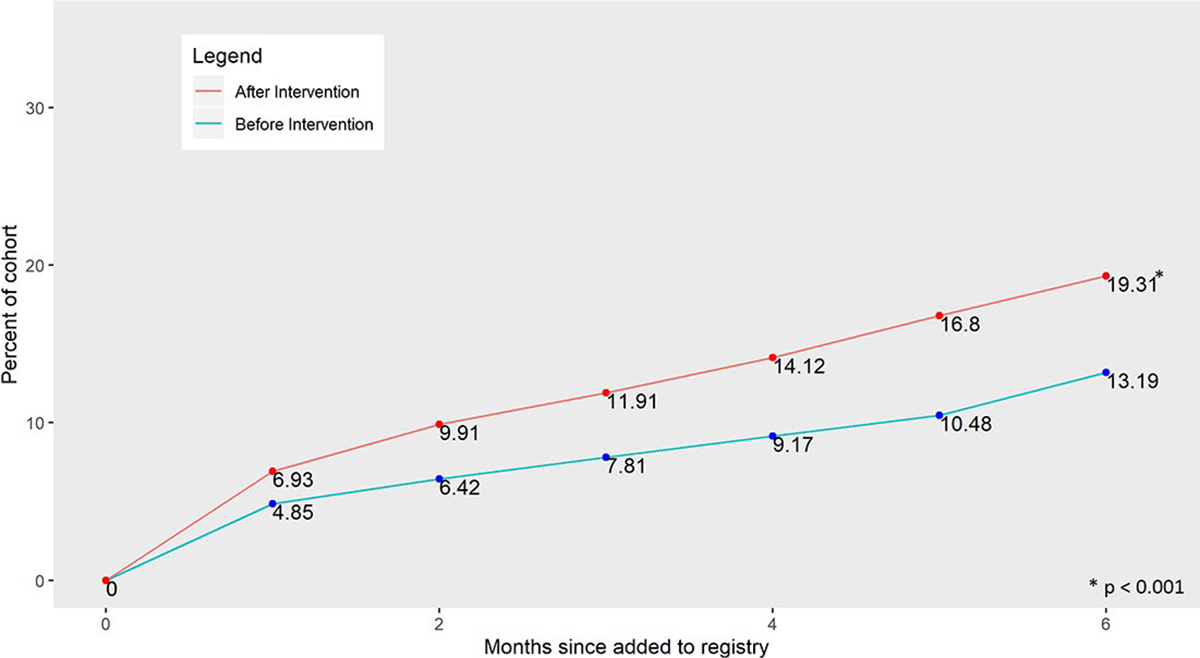
Cumulative spirometry referrals over time before the intervention vs. After the intervention.

**Figure 2.**
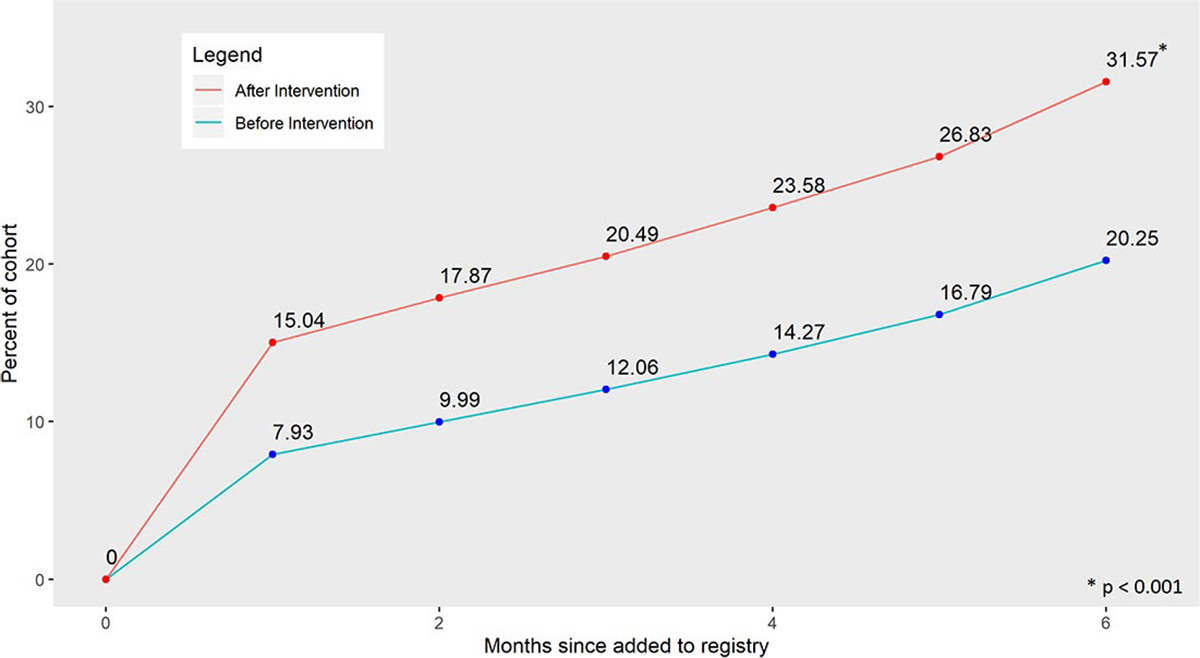
Cumulative spirometry completion rates over time before the intervention vs. After the intervention.

Between March 23, 2015 and September 24, 2018, there were 10,949 unique patients eligible for inclusion. 4,895 patients (44.7%) were excluded because they had completed spirometry prior to the cohort start dates. The pre-intervention cohort consisted of 2,662 patients (35.9% of all patients system-wide with a diagnosis of COPD and a primary care visit during that time period) and there were 3,392 patients in the post-intervention cohort (41.2% of all patients with a diagnosis of COPD and a primary care visit during that time period). Baseline demographic features for study participants are noted in Table 1. A greater proportion of patients in the post-intervention cohort were referred for spirometry (31.6% vs 20.2%, *p* < 0.001) and completed spirometry (19.3% vs 13.2%, *p* < 0.001) compared to patients in the pre-intervention cohort (Table 2, Figures 1 and 2). Among the patients who completed spirometry, discrete results were missing in 25 patients in the pre-intervention cohort and 33 patients in the post-intervention cohort. Chart review revealed these to be due to erroneous medical record number linkage or inability to complete testing in accordance with lab standards. Among those with spirometry results, 76 patients in the pre-intervention cohort and 65 in the post-intervention cohort did not have post-bronchodilator testing. When tabulating only completed results, there was a non-significant difference between the cohorts in the proportion of studies without evidence of airflow obstruction (58.3% vs. 63.5%, *p* = 0.07).

Among the 948 total patients in the study who underwent spirometry, 585 (61.7%) had no evidence of airway obstruction. A post-hoc interrupted time series analysis showed that the increase in spirometry referral was not related to prior secular trends in referral rates over time (supplementary material).

In a multiple variable analysis consisting of patients from both pre and post-intervention cohorts, patients were more likely to have no evidence of airway obstruction if they were female (Adjusted odds ratio [aOR] = 0.54, 95% Confidence interval [CI] = 0.41 – 0.72), were Black (aOR = 0.55, 95% CI = 0.40 – 0.75), had a BMI over 30 (aOR = 0.40, 95% CI = 0.30 – 0.54), or had comorbid conditions including cardiovascular disease (aOR = 0.79, 95% CI = 0.59 – 1.06) or sinus disease (aOR = 0.59, 95% CI = 0.39 – 0.88) (Table 3). A diagnosis of asthma, however, was associated with a greater likelihood of having airway obstruction (aOR = 2.10, 95% CI = 1.42 – 3.12).

Among the 1,016 patients who received messages *via* their patient portals to schedule their spirometry, 85 (8.4%) completed spirometry during the post-intervention period, and 61 (6.0%) scheduled the test on their own through the patient portal interface.

Inhaler prescriptions were examined between tested patients with and without evidence of airway obstruction. Among patients with airway obstruction without comorbid asthma, 44.3% were prescribed a long-acting inhaler prior to testing whereas only 25.8% of patients without evidence of airway obstruction were prescribed an inhaler prior to testing (*p* < 0.001). The majority of patients were prescribed either a long-acting muscarinic antagonist or an inhaled corticosteroid/long-acting beta agonist (Table 4).

## Discussion

In this before and after study of patients with a diagnosis of COPD who received primary care within an urban, safety-net hospital system, the implementation of three simultaneous EHR-based interventions across the system was associated with significant increases in the rates of both spirometry referrals and completions. These novel interventions were implemented systemically across a healthcare system over an extended period of 1.5 years. Testing uncovered a sizeable burden of overdiagnosis and potentially overtreated patients in our diverse patient population.

In keeping with data published from other studies, most patients in our cohort who underwent testing (61.7%) had no evidence of obstruction on post-bronchodilator spirometry and therefore were overdiagnosed [5, 15, 25]. At the individual level, the significance of a diagnosis of COPD in the absence of post-bronchodilator obstruction has been openly debated [26]. Smokers have been shown to have clinical symptoms, resource utilization and/or subclinical radiographic features associated with COPD, even when obstruction has been ruled out [27]. The management of such patients is invariably at the discretion of the treating physician, but there is a potentially greater risk of inappropriate prescription of long-acting inhalers in a way that is not otherwise guided by objective data and evidence-based indications. Importantly, overdiagnosis can lead to missed opportunities for the diagnosis of an alternative cause of a patient’s symptoms. The purpose of our intervention was to obtain spirometry when it was otherwise missing *after* the diagnosis of COPD was made, presumably based on symptoms and clinical presentation alone. Further work will be required to determine what the individual impact of either obstructed or non-obstructed spirometry results might be on these patients.

Overdiagnosis was more common among women, never smokers, the morbidly obese and those with a diagnosis of asthma or sinus disease, consistent with prior data [25]. In addition, we found that the likelihood of non-obstructed spirometry was higher in self-identified Black patients. The underpinnings and implications of this racial disparity warrant further evaluation, particularly through the lens of the likely misestimation of “normal” values based on self-identified constructs of race [28].

Our finding that patients who had non-obstructive spirometry were significantly less likely to have been on long-acting bronchodilators or inhaled steroids suggests that providers were not as confident in the diagnosis that was documented in the EHR. Many of these diagnoses may be erroneous and otherwise disregarded by primary care providers, but EHRs have an unfortunate potential to propagate such misdiagnoses [29]. Even then, our finding that 25.8% of non-obstructed patients in our cohort were prescribed therapy for COPD indicates that the number of potentially overtreated patients in our cohort was not trivial.

While many studies have highlighted the prevalence of COPD diagnoses without spirometry, our study is the first to evaluate a standardized clinical-decision support-based approach to addressing this gap. By leveraging the EHR and patient portal for this purpose, the process is integrated into standard clinical care and is automated, requiring little to no upkeep. At least from the provider’s perspective, such prompting has been shown to be helpful in closing numerous important care gaps [30–32]. Extending such “health maintenance” prompts to the patient portal can also boost gap closure [33]. While our study did leverage direct patient portal outreach, only 1,016 of 3,392 patients had an active patient portal and a fraction of these patients completed testing (85 of 1,016, or 8.4%). Most of these completions were self-scheduled (61 of 85, or 71.8%). The limited success of patient portal outreach in our unique patient population is unfortunately consistent with prior published reports [34].

More than half of our patients who underwent testing were overdiagnosed with COPD, and approximately one quarter of those without a diagnosis of asthma were potentially overtreated. Extrapolating our data to a national estimate of approximately 16 million individuals diagnosed with COPD suggests that there are potentially millions of overdiagnosed patients, hundreds of thousands of whom may be overtreated and could potentially be identified using approaches like ours.

The strengths of our study include the use of reproducible and pragmatic prompts in a diverse real-world patient population over an extended period of time. The generalizability of our study is limited by its reliance on a single site, non-randomized pre-post-design, and inability to specifically attribute the increase of spirometry referrals and completions to any one of the 3 EHR-based interventions that were implemented simultaneously.

### Interpretation

EHR-based interventions can increase spirometry referral and completion rates in patients with a clinical diagnosis of COPD who did not have confirmatory spirometry. Most potentially overdiagnosed patients who underwent spirometry were not obstructed, and up to 1/4^th^ of those without a diagnosis of asthma may be overtreated. Further research is warranted to understand the clinical implications of uncovering COPD overdiagnosis and its effect on overtreatment.

## Supplementary Material

Supplementary Material

## Figures and Tables

**Table 1.** Study cohort demographics.

	Pre-intervention cohort *N* = 2662	Post-intervention cohort *N* = 3392	*P*-value
Age (avg)	66.2	65.4	0.33
Sex (%)			0.99
Female	1490 (56)	1898 (56)	
Male	1172 (44)	1493 (44)	
Preferred Language (%)			0.63
English	2539 (95.4)	3240 (95.5)	
Spanish	70 (2.6)	78 (2.3)	
Other	53 (2.0)	74 (2.2)	
Race (%)			0.38
White	1718 (64.5)	2194 (64.7)	
Black	796 (29.9)	983 (29.0)	
Other	148 (5.6)	215 (6.3)	
Ethnicity (%)			0.0087
Hispanic	118 (4.4)	154 (4.5)	
Non-Hispanic	2469 (92.7)	3092 (91.2)	
Unknown/Declined	75 (2.8)	146 (4.3)	
Comorbidities (%)			
Asthma	823 (30.9)	866 (25.5)	< 0.001
Sinus Disease	906 (34.0)	1100 (32.4)	< 0.001
Cardiovascular	1623 (61.0)	1978 (58.3)	< 0.001

**Table 2.** Pulmonary function referral, completion and results.

	Pre-intervention Cohort	Post-intervention Cohort	P-value
**Spirometry Referrals**			
Total Number of Patients	2662	3392	
Referrals	539 (20.2)	1071 (31.6)	<0.001
Completed spirometry	351 (13.2)	655 (19.3)	<0.001

	Pre-intervention Cohort	Post-intervention Cohort	

**Spirometry Results**			
All results	351	655	
Discrete spirometry data missing* (%)	25 (7.1)	33 (5.0)	
Non-obstructed Result^†^ (%)	190 (58.3)	395 (63.5)	

* Due to inability to complete testing or erroneous medical record number linkages.

† Obstruction was defined as forced expiratory volume in one second (FEV 1) to forced vital capacity (FVC) ratio (FEV 1/FVC) less than the lower limit of normal (at the 5th percentile).

**Table 3.** Results of multivariable logistic regression.

	Non-obstructed*spirometry result (FEV1/FV*C* < 5% predicted value)	Obstructed*spirometry result (FEV1/FV*C* ≥ 5% predicted value)	P value	Adjusted odds ratio for obstructed result*	95% confidence interval
**Total**	585	363			
Sex					
Male	224	194			
Female	361	169	<0.001	0.54	(0.41, 0.72)
Age					
<50	76	54			
50–59	196	132	0.33	0.80	(0.50, 1.26)
60–69	172	114	0.53	0.86	(0.53, 1.38)
> =70	141	63	0.04	0.57	(0.33, 0.98)
BMI					
BMI < 30	265	236			
BMI ≥ 30	320	127	<0.001	0.40	(0.30, 0.54)
Race					
Caucasian	334	261			
Black	201	89	<0.001	0.55	(0.40, 0.75)
Other	50	13	0.05	0.45	(0.20, 0.96)
Ethnicity					
Non-Hispanic	526	345			
Hispanic	36	9	0.15	0.50	(0.19, 1.26)
Other	23	9	0.55	0.72	(0.29, 1.67)
Smoking History					
Current	308	221			
Former	209	129	0.56	1.10	(0.80, 1.52)
Never	63	11	<0.001	0.27	(0.13, 0.54)
Other	5	2	0.41	0.47	(0.06, 2.61)
Comorbidities					
Asthma	97	72	<0.001	2.10	(1.42, 3.12)
Sinus Disease	103	44	0.01	0.59	(0.39, 0.88)
Cardiovascular	278	145	0.12	0.79	(0.59, 1.06)

* Obstruction was defined as forced expiratory volume in one second (FEV1) to forced vital capacity (FVC) ratio (FEV1/FVC) less than the lower limit of normal (at the 5th percentile) following the administration of bronchodilators.

**Table 4.** Inhaler class comparisons in patients without a diagnosis of asthma.

	Non-obstructed spirometry result*N* = 488	Obstructed spirometry result*N* = 291
**Medication Class (%)**		
Any long-acting inhalers or inhaled steroids	126 (25.8)	129 (44.3)
LAMA	68 (43.9)	75 (44.1)
LABA	5 (3.2)	6 (3.5)
ICS	5 (3.2)	1 (0.6)
LAMA + LABA	3 (1.9)	2 (1.2)
ICS + LAMA	0 (0)	0 (0)
ICS + LABA	74 (47.7)	84 (49.4)
ICS + LAMA + LABA	0 (0)	2 (1.2)

* LAMA = Long acting muscarinic antagonist, LABA = long acting beta agonist, ICS = inhaled corticosteroid.

## ABBREVIATIONS

aOR: Adjusted Odds Ratio
BOLD: Burden of Obstructive Lung Disease Study
CI: Confidence Interval
COPD: Chronic Obstructive Pulmonary Disease
CPT: Current Procedural Terminology
EHR: Electronic Health Record
FEV1: Forced Expiratory Volume in one second
FVC: Forced Vital Capacity
GOLD: Global Initiative for Chronic Obstructive Lung Disease
ICD: International Disease Classification
ICS: Inhaled Corticosteroid
LABA: Long-acting Beta agonist
LAMA: Long-acting Muscarinic antagonist
OR: Odds Ratio
