# Supplementary material for: The Impact of an Electronic Health Record Intervention on Spirometry Completion in Patients with Chronic Obstructive Pulmonary Disease
Source: COPD. Author manuscript; Available in PMC 2022 Jun 16. (PMC9202241; doi:10.1080/15412555.2022.2049736)
Supplement: Supplementary Material [file NIHMS1809743-supplement-Supplementary_Material.docx]

Supplemental Material:

We conducted an interrupted time series analysis by analyzing monthly rates of referrals in patients with a diagnosis of COPD, but without PFTs 1 year prior to, and 1 year after the intervention (3/1/2017).

A segmented multiple variable linear regression was conducted with variables included for secular trends, post-intervention step-up in rate and post-intervention changes in trends. The results of the regression are presented below.


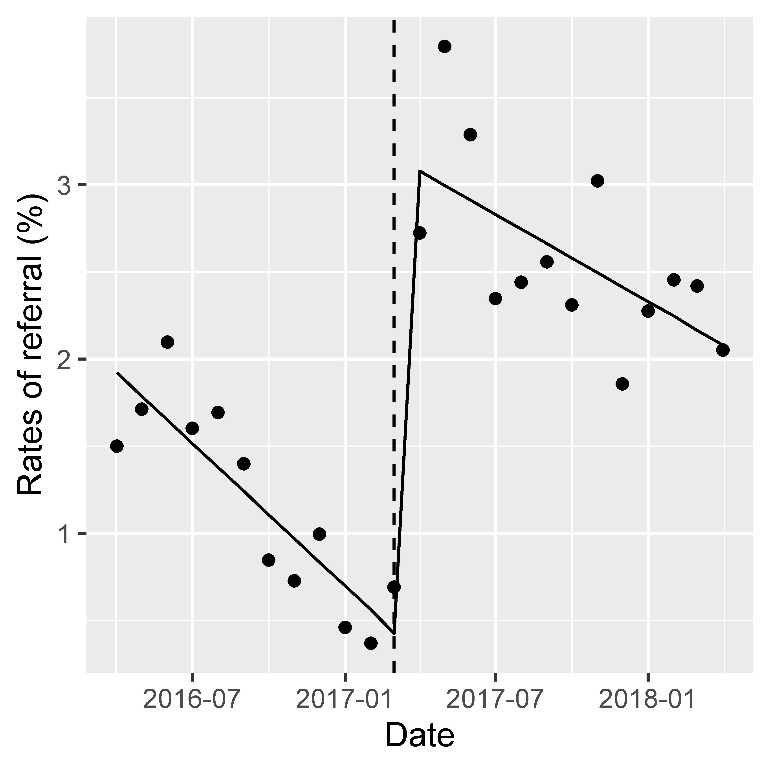


**Figure**: Interrupted time series analysis of rates of PFTs referral in eligible patients. The points are per-month rates of referrals, the dashed line references the point of initiation of the intervention and the solid black line shows the regression modeled estimates.

|  | Estimates | 95 % Confindence Interval | P-value |
| --- | --- | --- | --- |
| Overall trend | - 0.13 | -0.20 – (-0.072) | <0.001 |
| Post-intervention level change | + 2.79 | 2.17 - 3.41 | <0.001 |
| Post intervention trend change | + 0.05 | -0.032 – (-0.14) | 0.21 |

**Table:** Results of the interrupted time series analysis.
